# Effect of Ablation Depth on the Endothelial Status of Eyes of Myopic Patients Undergoing Transepithelial Photorefractive Keratectomy: A Retrospective Study in Saudi Arabia

**DOI:** 10.7759/cureus.64527

**Published:** 2024-07-14

**Authors:** Sultan H Alrashidi

**Affiliations:** 1 Department of Ophthalmology, College of Medicine, Qassim University, Buraidah, SAU

**Keywords:** central corneal thickness, ablation depth, corneal endothelial count, myopia, transepithelial photorefractive keratectomy

## Abstract

Purpose: The aim of this study was to correlate ablation depth on corneal endothelial cell (EC) indices following transepithelial photorefractive keratectomy (TPRK) in Saudi myopic patients.

Methods: This was a retrospective cohort study of myopic eyes treated with TPRK. The changes in EC density and other indices were noted one week (W1) and 12 weeks (W12) after TPRK, which was the primary outcome. The laser ablation depth (AD) was correlated to the EC indices. The preoperative factors were also correlated to the outcomes using regression analysis to review predictors of change in the EC density.

Results: We had 120 eyes of 60 myopic patients operated on for TPRK. The mean of maximum AD (Adm) and central AD (ADc) were 110.3±23.7 µ and 108.8±24.4 µ, respectively. The median change in EC count at W1 and W12 were -9.5 (interquartile range (IQR) -33.0, 17.0) and -3.0 (IQR -29.3, 13.3), respectively. The ADm was negatively correlated to a change in EC density at W1 (Wilcoxon (Z) =-2.7, P = 0.006) and at W12 (Z = -3.74, P <0.001). ADm (Kruskal-Wallis (K-W) test (P) = 0.167), ADc (K-W P = 0.08), central corneal thickness (K-W P = 0.65), and use of mitomycin-C (K-W P = 0.357) were not significant predictors of the change in EC density at W1. None of the variables significantly influenced the change in EC density at W12.

Conclusions: The corneal ablation depth for TPRK is correlated to changes in EC density at W1 and W12 after TPRK.

## Introduction

More and more of the young population opt for refractive surgeries for myopia [1,2]. Transepithelial photorefractive keratectomy (TPRK) as compared to other refractive surgeries has been claimed to be an efficient, safe, and more client-friendly procedure [3]. High-order aberrations, transient corneal haze, and residual refractive error are uncommon complications following TPRK [4,5]. Complications like corneal ectasia and corneal haze 20 years after photorefractive keratectomy (PRK) are rare, and therefore, this surgery is deemed safe in the long term [6].

Corneal endothelium comprises cells with 4-6 μm thickness and 20 μm in diameter and is of a hexagonal or polygonal shape. It can be studied in detail using confocal microscopy [7]. The indices for the corneal endothelium status include cell count, cell density, mean and standard deviations of cell size, coefficient of variation in percentage, maximum and minimum cell size, and cell hexagonality in percentage. Age and diabetes influence changes in corneal endothelium [8].

The corneal endothelium is a vital structure for the vitality of the cornea following refractive surgery. Exposure of endothelial cells (ECs) to ultraviolet rays through thin cornea while performing collagen cross-linkage has been found safe [9]. A study to evaluate the corneal endothelial toxicity PRK surgery with intraoperative use of mitomycin-C (MMC) found that EC count and mean cell had not significantly changed after surgery. However, corneal cell polymegathism and pleomorphism were noted post PRK with MMC usage [10].

The TPRK laser ablation of the cornea starts from the epithelial layer down to the anterior corneal stroma, and depth at the center and different points of the corneal surface are determined by the software incorporated in the machine. It could depend on the severity of myopia, astigmatism, irregularity on the anterior surface of the cornea, and overall corneal thickness [11]. These parameters include ablation depth (AD), total ablation zone (TAZ), transitional zone (TZ), and optical zone (OZ). The effect on corneal endothelium thus could be influenced by these surgery parameters and the duration of exposure to the laser rays [12]. To the best of our knowledge, the role of these surgery parameters on the status of corneal endothelium soon after and three months after TPRK has not been studied. 

We present the correlation of ablation parameters to endothelial indices before and after week one (W1) and week 12 (W12) follow-up of myopic eyes treated with TPRK and factors affecting the correlation of ablation parameters to changes in EC count after TPRK.

## Materials and methods

A retrospective evaluation of data from 60 myopic patients (120 eyes) who underwent TPRK surgery in a private ophthalmology institution, Vision Eye Specialist Center, Buraidah, Qassim, Saudi Arabia, was conducted between January and December 2022. All patients were operated on by a single surgeon. All processes were carried out according to the principles of the Declaration of Helsinki and its later revisions. Because of the retrospective study design, the need to obtain informed patient consent was waived.

Inclusion and exclusion criteria

Adults aged 18 years and more, with best corrected visual acuity (BCVA) 0.0 logarithm of the minimum angle of resolution (LogMAR) and likely to have a residual stromal bed of >350 μm at the thinnest location on the cornea and without dry eye scheduled to undergo TPRK during the study period were included in the present study. Patients with comorbidities like diabetes, autoimmune disease, pregnant females, and lactating women were excluded. Patients with a history of severe ocular trauma, keratoconus, corneal pathologies, and ocular surgeries in the past were excluded.

Sample size

We used a World Health Organization-recommended method for calculating the sample size to test a hypothesis in a cohort study [13]. To estimate the sample size for a one-armed cohort study, we hypothesized that the difference in the endothelial count at W12 after TPRK and before surgery was not significantly correlated to the maximum AD (ADm). The null hypothesis was the difference in EC count compared to before TPRK, which was significantly correlated to the ablation depth. To achieve a 95% confidence interval (CI) and 80% power to the cohort study of 1:1 ratio of eyes assessed before and after TPRK with anticipated 1.25 relative risk of EC damage in eyes, with 90% of eyes with good endothelial indices before surgery and 70% of eyes with good endothelial indices after surgery, we need at least 113 eyes to be reviewed. To compensate for the loss of patients for follow-up, we added 5% additional cases. Thus, the final sample for the present study was 120.

Measurements

The visual acuity for distance, both uncorrected and the best corrected, was assessed using a phoropter and Early Treatment Diabetic Retinopathy Study (ETDRS) chart. The LogMAR notations were used to document the visual acuity. Each eye's spherical, cylindrical, and spherical equivalent (SE) refractive status was measured in diopter (D). The corneal topography by a Pentacam® HR camera (OCULUS Optikgeräte GmbH, Wetzlar, Germany) and Sirius (SCHWIND eye-tech-solutions GmbH, Kleinostheim, Germany) enabled us to note keratometry (K1 and K2), central corneal thickness (CCT), and pupillary diameter. Spherical, cylindrical, and spherical equivalent (SE) refractive power in a diopter (D) were documented for each eye. The anterior segment of the eye was assessed using a slit-lamp biomicroscope (Topcon Corporation, Tokyo, Japan). The intraocular pressure was measured using an applanation tonometer mounted on the slit lamp.

The corneal EC-related indices were generated using non-contact specular microscopy (EM-3000; Tomey Corporation, Nagoya, Japan). The TOMEY EM-4000 has the feature of automatic and manual EC analysis named as Trace method, L-Count, Core Method, and Dark area analysis method. The mode of this assessment was a non-contact, auto-alignment, central fixation, and auto-measurement with the Trace method. Of the 16 images captured automatically, the best endothelial image was taken for the analysis. The procedures were repeated at W1 and W12 follow-ups after TPRK.

The detailed steps of TPRK are described in the literature [5,11,14]. For surface ablations, we used an Amaris 500 Hz excimer laser (SCHWIND eye-tech solutions GmbH). The machine was on aberration-free mode, with a 6.5-7.5 mm treatment zone, and adjusted as per the pupillary diameter to avoid uneven ablation. The purpose was to attain emmetropia. If the AD was more than 100 μm, we applied 0.3% MMC (0.2 mg/mL), diluted in balanced salt solution (BSS) on the ablated stroma for 30-50 seconds, then irrigation with BSS. The surgery was ended by the application of one drop of 0.3% ofloxacin and the application of a soft bandage contact lens.

Surgical parameters that were noted included duration of laser application, ADm (µm), central ablation depth (ADc) (µm), total ablation zone (TAZ) (mm), transitional zone (TZ) (mm), and optical zone (OZ) (mm).

All eyes postoperatively underwent a treatment regimen of 0.3% topical ofloxacin four times daily until the contact lens was in place, 0.1% dexamethasone drops four times daily for one week, followed by a gradual reduction of frequency in the next six to eight weeks, and artificial tears were instilled four times daily for 12 weeks.

The EC status-related indices at W1 and W2 after TPRK were compared to before TPRK. The EC count was the primary outcome. Other indices included cell density (cells/mm^2^), average (µm^2^), SD in µm^2^, coefficient of variation in percentage, cell count maximum, Cell count minimum, and hexagonality of EC (%). The difference in EC density before TPRK, at W1, and at W2 was calculated and used as the main outcome variable to correlate to the ablation depth.

Data analysis

The data was collected on a pretested data collection form. It was analyzed using IBM SPSS Statistics for Windows, Version 25.0 (Released 2017; IBM Corp., Armonk, New York, United States). The qualitative variables were presented as numbers and percentage proportions. The quantitative variables were plotted to study distribution. If the distribution was normal, we presented their mean and SD. We presented their median and interquartile range (IQR) if the distribution was skewed or the subsample was small. The endothelial indices at W1 and W12 compared to preoperative indices were validated using the nonparametric method, and the Wilcoxon signed rank test was carried out to estimate coefficient (Z) and two-sided P values. The Mann-Whitney U test assessed the two-sided P value for the qualitative variable as a dependent factor. The independent variables significantly correlated to the change in EC count at W1 and W12 were entered into a regression model using the step-out method to review the predictors of endothelial count differences. A p-value of <0.005 was considered statistically significant.

## Results

In this cohort, we evaluated 120 eyes of 60 Saudi myopic patients. The median age of the patients was 27.7±6.2 years (range, 19-46 years). There were 29 (48.3%) males. Surgery was undertaken on both eyes of each patient. Mild, moderate, and severe myopia grades were in 76 (63.3%), 39 (32.5%), and five (4.2%) eyes, respectively.

**Figure 1:**
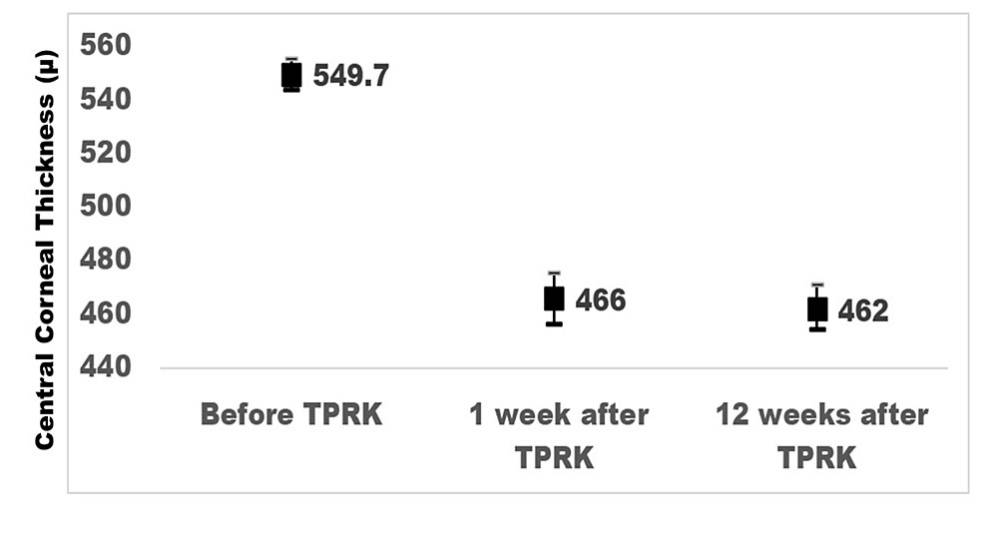
Comparison of central corneal thickness before TPRK and at one week and 12 weeks after TPRK to treat myopia. X axis denotes three periods for measurement of central corneal thickness; Y axis denotes central corneal thickness in (µ); Bar lines denote upper and lower end of 95% confidence interval of the mean of central corneal thickness TPRK: transepithelial photorefractive keratectomy

The preoperative ocular profile is compared to the post-TPRK ocular profile in Table 1. The visual target and emmetropia goals were attained in most of the operated eyes. The CCT was less than 400 µ at W1 in 17 (14.4%) and 13 (12.5%) eyes at W12 after TPRK (Figure 1).

**Table 1:** Ocular profile of myopic patients before and one week after TPRK TPRK: transepithelial photorefractive keratectomy; LogMAR: logarithm of the minimum angle of resolution

		Before TPRK	At 1 week post TPRK
Spherical equivalent refraction (Dioptre)	Median	-2.13	0
	Interquartile range	-3.75, - 1.50	-0.13, 0.38
	Range (minimum, maximum)	-7.75, 0.00	-1.75, 1.5
Spherical refractive error (Dioptre)	Median	-1.75	0.5
	Interquartile range	-3.25, -1.0	0.0, 0.75
	Range (minimum, maximum)	-7.0, 0.5	-1.75, 2.0
Cylindrical refractive error (Dioptre)	Median	-0.5	-0.5
	Interquartile range	-1.19, -0.5	-0.75, -0.25
	Range (minimum, maximum)	-3.75, 0.0	-2.75, 0.5
K_1_ (mm)	Mean	42.5	-
	Standard deviation	1.59	
	Range (minimum, maximum)	37.6, 43.8	
K_2 _(mm)	Mean	43.6	
	Standard deviation	1.6	
	Range (minimum, maximum)	42.6, 44.5	
Pupillary diameter (mm)	Mean	6.26	-
	Standard deviation	0.84	
	Range (minimum, maximum)	3.9, 7.9	
Uncorrected distance visual acuity (LogMAR)	Median	0.5	0
	Interquartile range	03, 0.8	0.0, 0.0
	Range (minimum, maximum)	0.0, 1.3	0.0, 0.0

The details of surgical indices for TPRK are given in Table 2. The distribution of excimer laser parameters was normally distributed and, hence, presented as the mean and SD. There were a few outliers, with maximum and minimum values varying widely.

**Table 2:** Transepithelial photorefractive keratectomy indices used to treat myopia

Indices		Values
Transitional Zone	Mean±SD (minimum, maximum)	1.14±0.29 (0.7, 2.0)
Optical zone	Mean±SD (minimum, maximum)	6.8±0.35 (6.3, 7.8)
Total optical zone	Mean±SD (minimum, maximum)	7.94±0.49 (6.6, 9.2)
Duration of laser (seconds)	Mean±SD (minimum, maximum)	56.0±14.6 (34, 109)
Ablation depth _Maximum_	Mean±SD (minimum, maximum)	110.3±23.7 (71, 185)
Ablation depth _central_	Mean±SD (minimum, maximum)	108.8±24.4 (70.8, 184.7)

The endothelial indices before TPRK and at W1 and W12 after TPRK are given in Table 3. Apart from the coefficient of variation (%) and hexagonality of ECs (%), the median of all other endothelial indices significantly declined at W1 compared to the preoperative values. At W12, maximum and minimum cell counts were not significantly different from before TPRK. All other endothelial indices differed significantly from those before surgery. In 54 (45.8%) eyes, EC density declined, and in 64 (54.2%) eyes, it increased at W1 compared to before TPRK. In 36 (34%) eyes, EC density declined, and in 70 (66%) eyes, it increased at W12 compared to before TPRK.

**Table 3:** Corneal endothelial indices before TPRK and at one week and 12 weeks after TPRK †  Wilcoxon signed rank test was used to estimate Z and two-sided P values for validation TPRK: transepithelial photorefractive keratectomy; IQR: interquartile range

	Before TPRK (n =120)		One week after TPRK (n = 118)			12 weeks after TPRK (n = 104)		
	Median	IQR	Median	IQR	Validation z (p)	Median	IQR	Validation†
Cell count	270	251, 292	262	240, 284	-2.2	266	240, 293	-1.94
					(0.03)			(0.05)
Cell density (cells/mm^2^)	2,852	2,725, 3,003	2,818	2,614, 2,966	-2.7	2,816	2,602, 2,967	-3.74
					(0.006)			(<0.001)
Average (micron meter^2^)	352	333, 367	355	337, 382	-2.59	356	338; 385	-4
					(0.01)			(<0.001)
Standard deviation in micron meter^2^	130	117.3, 145	129.5	119, 146	-1.1	135.5	120, 159	-4
					(0.28)			(<0.001)
Coefficient of variation in %	37	35, 40	37	34, 41	-0.62	38	35; 43	-3.3
					(0.53)			(0.001)
Cell count maximum	855	726, 1,005	904	754, 1172	-2.4	896	762, 1071	-1.34
					(0.02)			(0.18)
Cell count minimum	90	81, 101	94	82, 105	-1.7	90	80, 99	-0.93
					(0.09)			(0.36)
Hexagonality of endothelial cells (%)	49	44, 59	50	44, 55	-0.64	46	40, 52	-4.4
					-0.5			(<0.001)

Changes in EC density at W1 and W12 were correlated/associated with the preoperative and operative parameters (Table 4). The changes in endothelial density at W1 and W12 after TPRK were significantly correlated to the preoperative SE (Wilcoxon P = 0.001) and CCT (P <0.001). Both ADm (P <0.001) and ADc (P <0.001) were significantly and negatively correlated to endothelial cell density at W1 and W12. The duration of laser application on corneal stoma was not significantly correlated to the endothelial density at W1 and W12 after TPRK.

**Table 4:** Changes in endothelial cell density at one week and 12 weeks compared to before TPRK for myopia and determinants TPRK: transepithelial photorefractive keratectomy

Variables		One week after TPRK (n = 118)		12 weeks after TPRK (n = 104)	
		Z	Two-sided P	Z	Two-sided P
Age	Wilcoxon signed rank test	-1.22	0.224	-1.5	0.129
Gender	Mann-Whitney U test	-1.25	0.21	-1.82	0.07
Spherical equivalent refraction	Wilcoxon signed rank test	-3.2	0.001	-4.1	<0.001
K_1_	Wilcoxon signed rank test	-0.117	0.9	-0.85	0.4
K_2_	Wilcoxon signed rank test	-0.205	0.837	-0.75	0.45
Mitomycin C used	Mann-Whitney U test	-1.3	0.193	-0.18	0.858
Central corneal thickness	Wilcoxon signed rank test	-8.7	<0.001	-8.1	<0.001
Variables related to surgery					
Maximum ablation depth	Wilcoxon signed rank test	-4.1	<0.001	-3.5	<0.001
Central ablation depth	Wilcoxon signed rank test	-4.0	<0.001	-3.44	<0.001
Duration of laser	Wilcoxon signed rank test	-0.99	0.322	-0.07	0.94
Total ablation zone (mm)	Wilcoxon signed rank test	-2.43	0.015	-3.4	0.001
Transitional zone (mm)	Wilcoxon signed rank test	-3.0	0.003	-3.5	0.001
Optical zone (mm)	Wilcoxon signed rank test	-2.5	0.012	-3.5	0.001

ADm (K-W P = 0.167), ADc (K-W P = 0.08), CCT (K-W P = 0.65), and the use of MMC (K-W P = 0.357) were not significant predictors of the change in EC density at W1 after TPRK. However, preoperative SE (K-W P = 0.05) and duration of laser application (K-W P = 0.028) were significant predictors of positive and negative changes in EC density at W1 post TPRK.

At W12 after TPRK, the positive and negative changes in EC density could not be predicted by preoperative SE (P = 0.454), CCT (P = 0.226), use of MMC (P = 0.459), duration of laser exposure (P = 0.445), ADm (P = 0.231) and ADc (P = 0.340).

## Discussion

In this study, there was wide variation in the EC indices at different stages of assessments. In nearly half of the eyes, cell density declined at W1 and one-third at W12 after TPRK. AD was negatively correlated to the change in the EC density at W1 and W12 after TPRK. The depth of laser ablation in TPRK was not a significant predictor of an increase or decrease in the corneal EC density at W1 and W12 after TPRK.

Few studies suggest laser's negative impact on ECs in refractive surgery [15]. Exposure to the ultraviolet rays during collagen cross-linkage has not adversely affected the corneal endothelium concerning cell density and morphology, and over 12 months, the changes regress and functionally become normal [16]. The penetration and the toxicity of UV rays to endothelium would be different compared to the excimer laser used for stromal ablation and is likely to be more damaging to endothelium than UV rays. We did not find a study of the laser used for TPRK and its effect on the endothelium. The preferred refractive surgery for high myopia is Phakic intraocular lens implantation. It is more damaging to endothelium than corneal refractive surgery [17].

These living cells are closest and in the direct line of the laser beam when ablation is carried out on the corneal stroma. Based on the AD, this impact could vary. We noted increased corneal EC viability indices in some eyes undergoing TPRK, while others declined indices one week after surgical activities nearer to them. The increase in EC count and density suggests stimulation of EC proliferation. In contrast, the decline of these indices suggests toxic damage causing shrinkage and inflamed cell reactions. The changes do not seem to be sustained when all these indices were reviewed three months after TPRK, implying that all EC indices are transient and reversible [18].

Corneal haze is one complication that follows corneal stromal ablation. Various explanations are given [19]. They include keratocytes moving from the periphery to the ablated area, accumulation of glycosaminoglycans, and disorganization of lamellar layers of collagen in corneal stroma after TPRK [20]. MMC is recommended to manage corneal haze [21,22]. In addition, steroids are also helpful in reducing collagen activities post-laser ablation.

Age is a known factor influencing endothelial indices, and oxidative stressors mediate these changes [23]. However, these changes are pronounced in childhood and old age. Our cohort of myopic patients undergoing TPRK was young adolescents, and thus, age is less likely to be a confounding factor for noted changes in endothelial indices after surgery. Diabetes and glycemic control in patients with diabetes negatively influence corneal endothelium [24]. In our study, patients with diabetes were excluded; hence, these factors are not likely to be responsible for changes in endothelial cell indices.

There were a few limitations in this longitudinal study. At W1, one patient did not present for follow-up, and at W12, eight patients were lost to follow-up. The grades of myopia in these patients were mild, and the outcome of W1 was not systematically different. Hence, we believe they would have a similar result at W12 as those assessed. Although the status of stromal haze was not documented, all eyes had postoperative vision 0.0logMAR at W1. However, uncorrected visual acuity was not noted at W12 post TPRK. Thus, long-term haze and decreased vision cannot be ruled out.

Replicative senescence and stress-induced premature senescence are two molecular bases for the decreased proliferative capacity of corneal ECs [25]. Laser application on corneal stroma and subsequent exposure to medications without corneal epithelium might be stressors for endothelial cells. They could respond initially with increased indices followed by reduced cell number, density, morphology, and function for a limited period. In vivo, researchers have noted a positive, stimulating effect of insulin, growth factors (nerve growth factor, epithelial growth factor, fibroblast growth factor), bovine pituitary extract, ascorbic acid, serum, and various cytokines and mitogens [26]. Further research is recommended to study these factors on endothelium after TPRK.

## Conclusions

The variation in EC indices at W1 and W12 after TPRK was wide compared to those before surgery. The AD by excimer laser used in TPRK, although it influences some of the changes in indices related to the EC status after surgery, could not be a predictor. Preoperative refractive status and the CCT could determine AD. They should be accounted for before concluding the role of AD influence on EC-related indices after TRPK. 
